# The Role of Plastic Reconstructive Surgery in Surgical Therapy of Soft Tissue Sarcomas

**DOI:** 10.3390/cancers12123534

**Published:** 2020-11-26

**Authors:** Rebekka Götzl, Sebastian Sterzinger, Andreas Arkudas, Anja M. Boos, Sabine Semrau, Nikolaos Vassos, Robert Grützmann, Abbas Agaimy, Werner Hohenberger, Raymund E. Horch, Justus P. Beier

**Affiliations:** 1 Department of Plastic and Hand Surgery, Comprehensive Cancer Center, University Hospital of Erlangen, Friedrich-Alexander-University Erlangen-Nürnberg (FAU), 91054 Erlangen, Germany; s.sterzinger@web.de (S.S.); andreas.arkudas@uk-erlangen.de (A.A.); aboos@ukaachen.de (A.M.B.); raymund.horch@uk-erlangen.de (R.E.H.); jbeier@ukaachen.de (J.P.B.); 2 Department of Plastic Surgery, Hand and Burn Surgery, University Hospital RWTH Aachen, 52074 Aachen, Germany; 3 Department of Radiation Oncology, Comprehensive Cancer Center, University Hospital of Erlangen, Friedrich-Alexander-University Erlangen-Nürnberg (FAU), 91054 Erlangen, Germany; sabine.semrau@uk-erlangen.de; 4 Department of Surgery, Comprehensive Cancer Center, University Hospital of Erlangen, Friedrich-Alexander-University Erlangen-Nürnberg (FAU), 91054 Erlangen, Germany; nikolaos.vassos@umm.de (N.V.); Robert.Gruetzmann@uk-erlangen.de (R.G.); Werner.Hohenberger@extern.uk-erlangen.de (W.H.); 5 Division of Surgical Oncology and Thoracic Surgery, Department of Surgery, University Medical Center Mannheim, University of Heidelberg, 69117 Mannheim, Germany; 6 Department of Pathology, Comprehensive Cancer Center, University Hospital of Erlangen, Friedrich-Alexander-University Erlangen-Nürnberg (FAU), 91054 Erlangen, Germany; abbas.agaimy@uk-erlangen.de

**Keywords:** plastic surgery, soft tissue sarcoma, STS, reconstruction, reconstructive surgery

## Abstract

**Simple Summary:**

Soft tissue sarcoma (STS) are predominantly treated by surgery. Reconstructive surgery plays an essential role in interdisciplinary treatment of STS. In this study a 10-year single-center retrospective analysis of 290 SST patients revealed an association between clear surgical margin (R0) resections and higher-grade sarcoma in patients with free flaps with no significant differences in complication rates in regard to different reconstructive methods. Local recurrence risk was over two times higher with primary wound closure than with flaps. Defect reconstructions in STS are shown to be reliable and safe, thus lastic and reconstructive surgeons should have a permanent place in interdisciplinary surgical STS treatment.

**Abstract:**

Background: Soft tissue sarcoma (STS) treatment is an interdisciplinary challenge. Along with radio(chemo)therapy, surgery plays the central role in STS treatment. Little is known about the impact of reconstructive surgery on STS, particularly whether reconstructive surgery enhances STS resection success with the usage of flaps. Here, we analyzed the 10-year experience at a university hospital’s Comprehensive Cancer Center, focusing on the role of reconstructive surgery. Methods: We performed a retrospective analysis of STS-patients over 10 years. We investigated patient demographics, diagnosis, surgical management, tissue/function reconstruction, complication rates, resection status, local recurrence and survival. Results: Analysis of 290 patients showed an association between clear surgical margin (R0) resections and higher-grade sarcoma in patients with free flaps. Major complications were lower with primary wound closure than with flaps. Comparison of reconstruction techniques showed no significant differences in complication rates. Wound healing was impaired in STS recurrence. The local recurrence risk was over two times higher with primary wound closure than with flaps. Conclusion: Defect reconstructions in STS are reliable and safe. Plastic surgeons should have a permanent place in interdisciplinary surgical STS treatment, with the full armamentarium of reconstruction methods.

## 1. Introduction

Soft tissue sarcomas (STSs) are rare malignancies, and “up-to-date” sarcoma treatment remains challenging. Surgery plays the central role in the treatment of STS patients. Radiotherapy and chemotherapy are (neo)adjuvant treatment options that are often helpful. Historically, extremity sarcomas were often treated by radical surgery with limb amputation to achieve wide longitudinal margins. In the 1970s and 1980s, the paradigm slowly shifted to limb-conserving surgery [1]. Resection with negative margins is still mandatory in STS treatment, wherever possible [2,3]. STS resection may result in large or deep soft tissue defects and sometimes skin defects. Tissue reconstruction in STS, a classical domain of reconstructive surgery, can restore tissue, repair critical-size resection defects to cover crucial structures, restore function and salvage limbs [4,5]. Although limb salvage is only a secondary goal of tumor surgery, reconstructive surgery plays an essential role in sarcoma treatment because of the potential reconstructive benefits, while at the same time not adversely impacting tumor safety, survival or complication rates [6,7].

Due to the rarity of these malignancies, little is known about the impact of reconstructive surgery in the interdisciplinary treatment of STS. Most studies have focused on neoadjuvant and adjuvant radio(chemo)therapy and chemotherapy, although interest in reconstructive surgery in STS has continuously increased [4,6,8]. Reconstructive surgery can also allow an increased radiation dosage in recurrent tumors if nonirradiated tissue is transplanted. Furthermore, transplants may also help to reduce typical radiation sequelae such as fibrosis and radiation ulcers [9]. Nevertheless, the complication rates of reconstructive surgery in STS compared to surgical treatment of STS without reconstructive surgery have not been investigated thus far. Additionally, it is not sufficiently clear whether reconstructive surgeons’ usage of flaps, including microvascular free flaps, may be associated with increased numbers of free resection margins and thus with the success of STS resection.

Here, we analyzed the 10-year experience of the interdisciplinary surgical treatment of STS at a university hospital’s Comprehensive Cancer Center and focused on the role of reconstructive surgery. We performed a retrospective analysis of 290 patients’ medical records and evaluated those patients’ demographic and tumor characteristics, their survival rates and the impact of different reconstruction methods, and the complications and recurrence rates.

## 2. Materials and Methods

We performed a retrospective review of all patients who underwent surgical therapy for STS from 1 August 2004 to 31 July 2014, in surgery departments at our university hospital. The inclusion criteria for this analysis were a histologically confirmed diagnosis of STS and treatment in a surgical department at our university hospital. Patients with gastrointestinal stromal tumors (GISTs) were excluded, as were patients without a diagnosis of STS per the final tumor pathology report.

Data including demographics (i.e., age and sex); sarcoma diagnosis (i.e., first diagnosis date, tumor classification (TNM), tumor grade and recurrence status); tumor location (upper extremity, lower extremity, trunk and head/neck); management history of treatment including outside institutions; history of operative interventions; history of postoperative complications; use of neoadjuvant or adjuvant therapy; operative details of reconstruction; classification of resection margins using the residual tumor classification system (R0, complete resection; R1, microscopic residual tumor and R2, macroscopic residual tumor); follow-up and postoperative complications were extracted by reviewing the digital medical records.

Major complications were defined as complications necessitating an operative or in-hospital treatment (Clavien-Dindo ≥ 3) [10]. The numbers of operations and in-hospital treatments were also analyzed (1, 2, 3 and >3). We divided our patient cohort into three different groups of complications: “major complications after plastic surgery reconstruction” (complications that are directly linked to the plastic surgery reconstruction procedure), “major complications of sarcoma resection” (complications that are linked not to the plastic reconstruction but to the tumor resection procedure) and “total major complications” (the sum of the two complication rates defined above).

Patients were divided into subgroups undergoing (a) primary wound closure or plastic surgery reconstruction with (b) split skin grafts, (c) local/regional flaps or (d) free flaps.

Calculations were performed using the Statistical Package for the Social Sciences (version 19.0, SPSS Inc., Chicago, IL, USA). Survival was assessed in relation to possible influencing factors using the Kaplan–Meier method with log-rank tests (Mantel–Cox). Multivariate analysis of survival was assessed with Cox regression. Comparison of patient characteristics between the groups was performed by cross-tabulation and the chi-square test for categorical variables, the Mann–Whitney U test and Fisher’s exact test for ordinal variables, and Student’s *t*-test for continuous variables. The study was conducted in accordance with the Declaration of Helsinki. Each patient had signed an IRB approved informed consent form to be registered in the sarcoma database of the Comprehensive Cancer Center (CCC) prior to treatment at the CCC (no separate IRB approval for this specific retrospective analysis/retrieval of these medical record data necessary).

## 3. Results

We identified a total of 290 patients treated from 2004 to 2014. Of these, 280 underwent sarcoma resection in the Department of Surgery or the Department of Plastic and Hand Surgery. Eighty-one patients underwent initial sarcoma resection in other different hospitals without specific oncologist expertise for the STS; of these, only seven had an R0 status and had received reconstructive surgical treatment exclusively at our institution. The patients’ demographics and tumor characteristics are shown in Table 1. The most common localization of the STS in our cohort was the lower extremity (46.6%), followed by the trunk (19%); inside the abdomen or thorax (18.6%), with involvement of the thoracic/abdominal wall in four of these cases; the upper extremity (12.1%); and the head/neck (3.8%). Among tumor entities, liposarcoma was the most frequent, followed by fibroblastic/myofibroblastic sarcoma. The subtype distribution and localization of the sarcomas treated at our institution are also illustrated in Table 1.

A total of 168 patients underwent primary wound closure, while 22 patients were treated with split skin grafts. Local/regional flaps were used in 54 patients, and 40 patients received free flaps. Six patients were excluded from the reconstruction analysis because of early termination of operative treatment during the hospital stay due to advanced tumor disease. Twenty-eight percent (*n* = 81) of all included patients underwent initial STS resection in an external institution; of these, 58% underwent R1 resection in other different hospitals without an oncologist for the STS. Furthermore, 20% had a macroscopic residual tumor (R2) and 13% had unclear resection margins (RX; Table 2). In the case of positive (R1 and R2) or unclear (RX) margins or minimal free margins (total *n* = 71), a second operation in our institution was performed.

**Figure 1:**
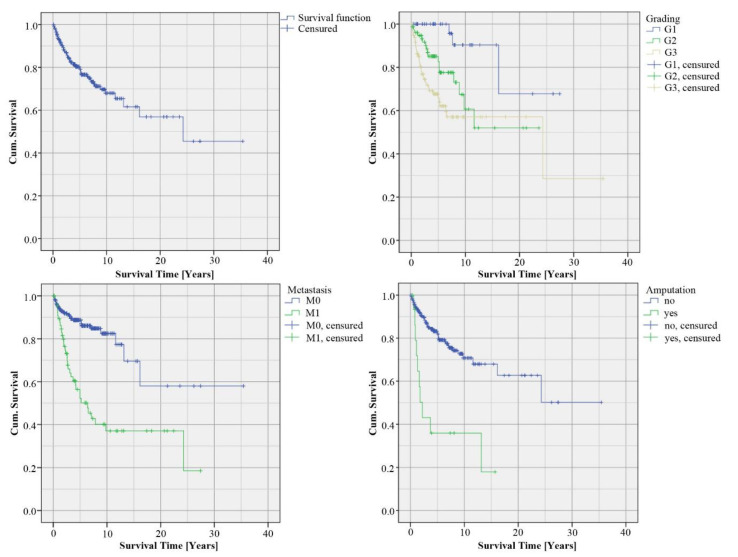
Kaplan–Meier curves of cumulative survival.

The overall survival rate was 79%. The Kaplan–Meier curve included 286 patients, of which 64 patients died during a mean follow-up period of 48 months, 145 cases were censored and 77 patients were lost to follow-up and were therefore censored at their last contact with the clinic. The majority of the tumors were high-grade STS (G1: 19%, G2: 36% and G3: 45%). The 5-year survival analyzed based on the grading was for G1 100%, for G2 78% and for G3 66% (*p* = 0.001). The 10-year survival was 90% of G1, 61% of G2 and 57% of G3. Furthermore, we analyzed survival time depending on the occurrence of metastasis. In our cohort, 76% of the patients had no metastasis (M0), while in 34% of the cases, metastasis was detected (M1). The 5-year survival of M0 patients was 89% (vs. 52% M1, *p* < 0.001) and the 10-year survival was 82% for M0 (vs. 37% M1, *p* < 0.001). We analyzed the survival rate of patients with limb amputation (*n* = 17, 6% of all cases). The 5-year survival of the patients with limb amputation was 36% (vs. 82% without amputation, *p* < 0.001 (Figure 1), univariate Cox regression with hazard ratios and 95% CI see Table 3). 

We focused further on surgical management regarding the different reconstruction modalities. Therefore, we divided our cohort into four groups: primary wound closure, use of split skin grafts, local/regional flaps or the use of free flaps with microvascular anastomosis. Analysis of the different groups regarding age, sex or recurrence situation retrieved a few significant differences (Table 4). Median age was significantly higher in the group of split skin grafts vs. primary wound closure, significantly more men were in the group of local/regional flaps vs. primary wound closure, and the sizes of tumor excision were significantly different in the groups of split skin grafts and local/regional flaps vs. primary closure, but not in the group of free flaps with microvascular anastomosis.

Significantly more R0 resections were associated with free-flap reconstruction (*n* = 39, 97.5%) compared to primary wound closure (*n* = 133, 79% R0 resection, *p* = 0.02). The tumor grading of patients with reconstruction using free flaps with microvascular anastomosis was significantly higher (free flaps: G1 6.5%, G2 48.8%; primary wound closure: G1 25%, G2 31.1%; *p* = 0.035).

**Figure 2:**
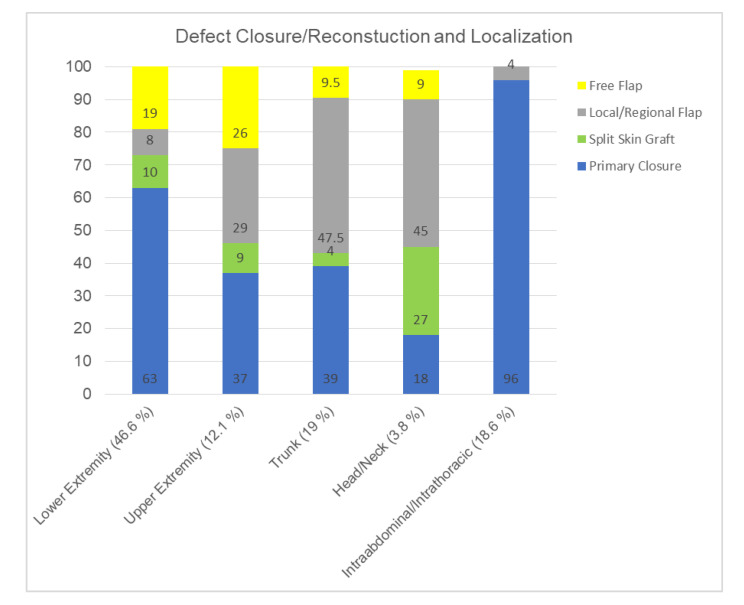
Overview of the percentages of defect/tissue/function reconstruction types depending on the localization.

Depending on the anatomic particularities, we observed different frequencies of reconstruction regarding STS localization. An overview of the percentage of type of reconstruction depending on the localization is given in (Figure 2). Patients with STS at the head and neck had the highest rate of reconstruction (including split skin grafts) compared to primary closure. In the case of STS at the extremities, free flaps with microvascular anastomosis were performed in 19 (lower extremities) and 26 (upper extremities) cases. The highest number of reconstructions was performed in cases of lower extremity STS (*n* = 50). In four patients with abdominal/thoracic STS reconstruction pedicled vertical rectus abdominis myocutaneous flap (VRAM-flaps) were performed.

To further assess the impact of the surgical therapy, we analyzed the complication rates (Table 5). The differences in total complication rates between the variant reconstructions were statistically significant (split skin graft: 19%, local/region flaps: 36% and free flaps: 31%) compared to primary wound closure. Total major complications were the lowest if primary wound closure was possible (9.5%). Further analysis of complication rates after sarcoma resection or reconstruction showed no significant differences (Table 5).

We further analyzed the type of major complications after plastic surgery reconstruction, which is shown in Table 6. After split skin grafts, significantly more additional free flaps with microvascular anastomosis were necessary than after local/regional flaps (22.7% vs. 5.6%, *p* = 0.041). Partial flap loss was not significantly different in the group of free flaps vs. local/regional flaps (7 vs. 4 cases, 17.5% vs. 7.4%, Fisher exact test: *p* = 0.195) and rendered most times into conservative wound therapy, only two patients needed an additional free flap. Loss of free flap (10%) was caused by arterial and venous thrombosis (7.5%) and in one case by an extensive partial loss because of in-flap perfusion problems, which finally lead to a complete flap loss (Table 6).

For analyses, we examined part of our cohort, which was treated with neoadjuvant radiochemotherapy (RCT) and adjuvant radiotherapy (RT; 47% vs. 10%). After neoadjuvant RCT, 28% of the patients had major complications, compared to 11% (no neoadj. RCT, *p* < 0.001), and after adjuvant RT, 15% had major complications, vs. 20% (no adj. RT, *p* = 0.004).

Furthermore, we evaluated patients’ age impact on major complication rates. No statistically significant influence was detected (Table 7).

In addition, we evaluated the impact of the timepoint of reconstruction after sarcoma resection on the complication rates. Therefore, we analyzed the major complications associated with reconstruction. We differentiated between a single-stage reconstruction (at the timepoint of sarcoma resection) or a two-stage reconstruction (normally with the use of topical negative pressure therapy in between). In our cohort, of 112 patients who underwent plastic reconstruction, 54 patients received a single-stage reconstruction at the timepoint of sarcoma resection. The other group of two-stage reconstruction (*n* = 58) was divided further into an early-secondary (up to 30 days after sarcoma resection; *n* = 49) and a late-secondary (more than 30 days after sarcoma resection, *n* = 9) group. In the group of late-secondary reconstructions, we measured a complication rate of 56% (vs. single-stage: 28%, and early-secondary: 27%), which was not statistically significant. We also did not observe a higher R1 resection rate in single-stage reconstructions versus secondary reconstruction (11%, vs. 13%, not statistically significant).

In the group of patients with plastic surgery reconstructions, 18 patients were treated for STS recurrence, while the other 76 patients were treated for primary STS diagnosis. Operative treatment of STS recurrence was connected with a statistically significant higher wound-healing disorder rate of 39% (vs. 11% primary diagnosis, *p* = 0.008).

Furthermore, we evaluated a possible learning and training effect in the operation technique of local/regional pedicled flaps versus free flaps with microvascular anastomosis. Therefore, we divided the 10-year study period into two periods of five years (2004–2009 and 2010*–*2014). In the first 5 years, 34 local/regional flaps and 17 free flaps in patients with STS were operated on. In the second period, 20 local/regional flaps and 23 free flaps were performed, which were an increase of 33%, respectively 53% in the case of free flaps. The complication rate decreased in local/regional flaps from 37% in the first five years to 32%, and in cases of free flaps from 41% to 22%. However, the effects were not statistically significant.

In addition, we analyzed the rate of patients with tumor recurrence within the different groups of wound closure and reconstruction (Table 8). In our cohort, 19% of the patients with primary wound closure suffered a local recurrence. This was significantly higher compared to the group of local/regional flaps (7%, *p* = 0.05) and compared to the patients with free flaps with microvascular anastomosis (5%, *p* = 0.03).

Multivariate analysis of recurrence (Tukey post-hoc test) showed no significant influence of grading, amputation, R-status or size of excision (Table 9). Multivariate analysis of survival (Cox regression) revealed only metastasis as the independent prognostic factor of survival (Table 9). Multivariate analysis (Tukey post-hoc test) of total major complications showed no significant influence of grading, reconstruction/closure method, R-status or size of excision (Table 9).

## 4. Discussion

In this study, we analyzed the 10-year experience of the interdisciplinary surgical treatment of STS in our University Hospital’s Comprehensive Cancer Center with a focus on the role of reconstructive surgery. The retrospective analysis of 290 patient medical records showed significantly more R0 resections in cases with free-flap reconstructions. Furthermore, the high tumor grade in patients who underwent reconstruction using free flaps with microvascular anastomosis was significantly higher. According to the varying localizations and the localization prevalence, patients with head and neck STS needed reconstruction most frequently (82%, *n* = 9). The highest absolute number reconstructions were performed in cases of STS at the lower extremity (37%, *n* = 50). Total major complications were lowest if primary wound closure was possible. Comparing the reconstruction techniques with primary wound closure, split skin grafts, local/regional flaps and free flaps had significantly more total major complications. Median age was significantly higher in the group of split skin grafts. After defect reconstruction with split skin grafts, more secondary free flaps with microvascular anastomosis were needed, followed by pedicled local/regional flaps. Notably, the age of patients or time point of reconstruction had no significant influence on the complication rate. In contrast, wound healing was impaired in the reconstruction in patients with STS recurrence. In our study, the risk for local recurrence was in the group with primary wound closure and was significantly (more than two-fold) higher than in the group with local/regional flaps or free flaps with microvascular anastomosis.

Surgery plays a central role in the treatment of STS patients. Radiotherapy and chemotherapy are (neo)adjuvant treatment options and are often helpful in STS, and in hard issue sarcomas [11,12]. Many studies in the literature identified positive margins as the main predictor for local recurrence [2,13]. In the case of large, deep and high-grade sarcomas with invasive growth into critical structures, including major nerves, vessels and bone, higher rates of metastasis are observed more frequently [14,15]. In our patient cohort, we found significantly more cases with free margins in the group with free-flap reconstructions. Additionally, local control was statistically significant more than two times higher in the group with local/regional flaps or free flaps than in the group with primary wound closure. However, in our cohort local recurrence rate was very high in cases with split skin grafts, even though the R0 rate was superior to primary closure. The local recurrence rate in cases of primary closure and split skin grafts was not significantly different. Multivariate analysis identified that neither grading, amputation, resection margins or size of excision are independent risk factors for recurrence in our cohort. Surprisingly we found a higher local recurrence rate in the split skin graft group, which seems inconsistent to the statement, that positive margins are the main predictor of local recurrence. Nevertheless, large studies in the literature investigating the prognostic significance of surgical margins in STS have also inconsistent results [2,16,17,18,19,20]. The reason for the high recurrence rate in cases with split skin graft remains unclear. However, a potential reason could be the margin widths especially to the depth, which maybe were sufficiently wide to achieve a split skin graft suitable wound bet without free critical structures. Nevertheless, in the literature close and wide negative margins did not seem to influence the local recurrence rates significantly [2,21], so a bias caused by the retrospective nature of our study could also be the reason for our observation.

In this study, grading was significantly higher in the group of free flaps compared to primary wound closure. In a study by Dadras et al., the same association of grading with primary wound closure compared to a group with flap transplantation was reported [5]. However, even with more high-grade sarcomas in our cohort, we observed significantly more cases with free margins in the group of patients with free flaps. This could indicate that sarcoma resection may be performed with a higher rate of free margins, i.e., sufficiently radical, when reconstruction options are provided by a separate team, i.e., plastic surgery, and therefore the oncological surgeon is not hampered by the morbidity of the defect and the subsequent challenge of reconstruction while performing the tumor resection.

However, some authors stated that the need for free flaps in STS reconstruction indicates failure in planning surgical management [22]. In our 10-year experience described here, we strictly followed the decisions of a multidisciplinary tumor board following established internal, evidence-based guidelines. The need for a microsurgical free flap was not related to failed planning of surgical management but to the given sarcoma resection defect. Since microsurgical free-flap reconstruction in a high-volume center does not come along with higher revision, major complication or flap loss rates than any other kind or reconstructive surgery, free flaps often pose the best, i.e., most efficient and effective means for reconstruction of complex resection defects. At our University Hospital’s Comprehensive Cancer Center, the whole reconstructive spectrum is available, which allows even extensive resections and complex reconstructions. Detailed preoperative surgical resection planning estimates the expected defect and the specific need for flap reconstruction [8,23]. Our interdisciplinary approach included a comprehensive preoperative discussion about possible intraoperative decision making in terms of defects so that more than one reconstructive option was always planned ahead of the operation. Possibly free microsurgical flaps should be offered and thus used more frequently in STS reconstruction because the surgical oncologist may feel no restriction in oncological resection, which could result in more frequently achieved R0 situation while coming along with more significant tissue defects. Reconstructive surgery can optimize the results, and in terms of soft and bone tissue and functional reconstruction, it is essential in the treatment of STS. According to this, other investigations also described the credits of microsurgical free flaps in STS reconstruction regarding functional outcomes and quality of life [24,25].

Flap selection (free flaps and local/regional flaps) was based on individual parameters such as tumor size and expected defect size, localization, patient positioning, anesthesia risk profile and functional restrictions. Size of excision was in our study not significantly different in the group of free flaps compared to primary wound closure. Surgeons’ individual preferences seemed to play a minor role, although this bias cannot be excluded due to the retrospective character of this study. However, analysis of differences between individual surgeons and type of reconstruction (and/or complication rates) would have been interesting. Due to very small subgroups, statistical analysis to investigate this parameter was not possible. As free flaps, fasciocutaneous or perforator flaps (e.g., anterolateral thigh perforator flap, ALTP) and muscle flaps with or without a skin paddle (e.g., latissimus dorsi flap) were used, if necessary. Analysis of further subgroups in the group of free flaps was not performed here because of small numbers per subgroup. Free flaps in STS have been discussed since 1993, and the overall success rate was 97% [26]. Similar studies demonstrated that free flaps are safe to perform in STS reconstruction [27,28,29,30]. Conversely, local and regional flaps subsumed more heterogeneous flaps. On the one hand, pedicled regional perforator (e.g., ALT) or muscle (e.g., vertical rectus abdominis myocutaneous flap, VRAM) flaps, and on the other hand, local flaps, such as rotation or transposition flaps, were performed. In our study and the literature, pedicled flaps were utilized most commonly [8]. Some areas of the body, i.e., the trunk, have more locoregional reconstructive options than others, such as defects at the head or neck. Correspondingly, the probability of using free flaps was relatively highest at the head and neck because of specific anatomic features of this body region [31]. Among the intraabdominal/intrathoracical sarcoma patients, a few suffered from abdominal/thoracic wall infiltration. This entity otherwise differs from STS outside the body cavities, since growth and tumor characteristics are different, and most resection only very rarely results in soft tissue defects [32]. However, in four of those patients, abdominal/thoracic wall involvement resulted in significant skin soft tissue defects, making reconstruction with pedicled vertical rectus abdominis myocutaneous flap (VRAM-flaps) necessary. Therefore, this subgroup was included in our analysis. Compared to the literature, the proportion of plastic surgery reconstruction itself was relatively high (40% of all cases) in our collective. In a study by Cannon et al., reconstructive surgery was performed in 20% of all STS patients [33]. In contrast to our results, López et al. concluded that free flaps were used more frequently at the lower extremity than in other parts of the body [34]. Kang et al. compared flap reconstruction to primary wound closure in extremity STS in a case-control study and found lower functional scores, higher wound complication rates and longer hospital stays but better local control in the flap group. Compatible with our results, their subgroup analysis of the flap group (local, regional and free flaps) showed no differences in the complication rate based on the type of flap [35].

In accordance with other publications on this topic, our study also has some limitations [36]. First, this is a retrospective analysis with different tumor localizations, different tumor statuses, various STS subtypes, and different reconstruction modalities. Therefore, statistical analysis is limited to sufficiently large, summarized and heterogeneous groups with varying values for the abovementioned parameters. Analysis of every single, stratified subgroup would have resulted in very small group sizes, thus rendering statistical analysis impossible. Furthermore, there are well-known limitations of a retrospective analysis per se, such as limited medical data records, especially concerning patients who decreased or lost follow-up and selection bias [37]. Future prospective studies with stratification regarding tumor size, grading, reconstruction modalities, resection margins and other here discussed parameters, and larger patient counts might result in more clear results. However, due to the infrequent number of these tumor entity limitation will always be the relatively low number of patients included, in particular when compared to far more frequent cancers like breast or colorectal cancer.

Nevertheless, complication rates between the reconstruction techniques were not significantly different between microsurgical free flaps, local/regional flaps or split skin grafts, but significantly higher compared to primary closure. It is known that microsurgical reconstruction after sarcoma surgery is reliable and safe [38,39], and many authors favor the use of microsurgical flaps over local options with regard to functional outcome and complication rates [6,27,38]. Complication rates themselves were in accordance with the literature [40]. However, after the use of split skin grafts, the need for additional microsurgical free flaps was significantly higher. We observed a high number of partial loss in the group of free flaps. We included all forms of partial loss (7 flaps, 17.5%), even little margin/tip of the flap-necrosis was accounted to partial loss. In most cases, only conservative treatment was needed, since only superficial tissue of most of these tip necrosis were affected. So, in other surgery studies this would have been summed up under “minor complications” (i.e., no operative therapy necessary) only. Compared to the group of local/regional flaps partial loss was not significantly higher (7 flaps vs. 4 flaps, *p* = 0.195).

In our study, patients treated with split skin grafts were significantly older (median age were higher), which could also have an effect on wound healing. Additionally, the frequencies of split skin grafts were different depending on the location. It is possible, that the localization itself is a risk factor of split skin failure. By contrast, in our cohort only eleven STS at the head and neck were included, so statistical analysis of this subgroup is impossible. Split skin grafts itself were only performed on tissues with vascular richness by surgeons of the Department of Surgery and of the Department of Plastic and Hand Surgery according the medical standard. In our experience, the use of split skin grafts (instead of local/regional or even free flaps) in sarcoma reconstruction should be weighed very critically. Split skin grafts may be the first option in cases of superficial skin defects. However, sarcoma therapy often includes neoadjuvant or adjuvant radio- or chemotherapy. Besides other possible causes of split skin graft failure described above, reconstruction with split skin grafts can lead to an unstable scar, and adjuvant radiotherapy may result in secondary breakdown of the grafted skin area. It would have been interesting to evaluate whether split skin grafts were used only in cases without a previously planned radiotherapy, but this was not possible due to inconsistent data regarding this question in the majority of medical records. Furthermore, in the case of local recurrence, wound complications were significantly higher than in the first resection.

Wound-healing disorders can be affected by many factors. A previous study showed higher complication rates after neoadjuvant RCT than in non-RCT in extremity STS [41]. In agreement with the literature, we also observed higher major complication rates after neoadjuvant RCT. It is likely that patients with STS recurrence had higher RCT rates or higher numbers of operations than those without recurrence, which probably also affects the wound complication rate [41].

Additionally, we examined the age of the patients or time between STS resection and reconstruction associated with higher complication rates. Decision making of single-stage or two-stage reconstructions, and early-secondary or late-secondary reconstruction, was performed individually patient-based. Single-stage reconstruction was performed in all cases with implants such as orthopedic prostheses or intraabdominal/intrathoracic meshes or if vulnerable anatomical structures such as major blood vessels or nerves were exposed upon tumor resection. In all other cases, if possible, histopathology results demonstrating free margins were awaited first before reconstruction was performed. In order to avoid infections or long hospital stays, reconstructions were performed as soon as possible after tumor resection. Late-secondary reconstruction was not performed unless unexpected medical conditions of the patients made postponing the reconstructive surgery mandatory. Based on this resection and reconstruction strategy, we did not observe significant differences in resection margins. In a 2019 meta-analysis of complications, reoperations and risk factors, Slump et al. identified lower limb tumors, diabetes, smoking, obesity and radiation as independent predictors of wound complications in extensive extremity STS [42]. In contrast, Dadras et al. reported similar patients’ characteristics, including age of patients with primary closure versus flap closure [5]. Lawrenz et al. reported that wound complication rates and oncologic outcomes remain similar, regardless of timing for STS reconstruction [43].

## 5. Conclusions

Interdisciplinary surgical STS treatment remains a challenging issue at present, and surgical resection often results in defects that need reconstruction, wherefore STS reconstruction requires a reconstructive plastic surgeon that can cover the full spectrum of reconstructive options. Using STS reconstruction methods, which are reliable and safe, microvascular free flaps may increase recurrence-free survival in large sarcoma resections because of an increased rate of negative resection margins. Hence, reconstructive surgery, including the full armamentarium of reconstruction methods, should have a permanent place in the interdisciplinary surgical treatment of STS because anatomical and functional reconstruction is essential in STS treatment.

## Figures and Tables

**Table 1:** Characteristics of the 290 included patients.

Patient/Treatment Characteristics	Values/Numbers		
Median Age (Years)	57.5 (7.4–89.1)		
Sex	Male	167	57.6%
	Female	123	42.4%
Neoadjuvant RT	Yes	136	46.9%
	No	154	53.1%
Neoadjuvant CT	Yes	141	48.6%
	No	149	53.1%
Adjuvant RT	Yes	31	10.7%
	No	259	89.3%
Adjuvant CT	Yes	44	15.2%
	No	243	83.8%
	Adjuvant, before reconstruction	3	1%
Localization	Lower extremity	135	46.6%
	Upper extremity	35	12.1%
	Trunk	55	19%
	Head/neck	11	3.8%
	Intraabdominal/intrathoracic	54	18.6%
STS Subtypes	Liposarcoma	74	25.5%
	Undifferentiated pleomorphic sarcoma	78	26.4%
	Fibroblastic/myofibroblastic sarcoma	51	17.6%
	Leiomyosarcoma	29	10%
	Rhabdomyosarcoma	16	5.5%
	Synovial sarcoma	14	4.8%
	Extraskeletal chondro-/osteosarcoma	12	4.1%
	Malignant peripheral nerve sheath tumor	10	3.4%
	Angiosarcoma	3	1%
	Other unclassified sarcoma (fibrohistiocytic and pericystic)	3	1%

**Table 2:** Tumor recurrence and metastasis status and resection margins of the 290 included patients.

Resection Status	Recurrence and Metastasis Status		
Recurrence/Metastasis Status at Timepoint of Resection (*n* = 288)	First diagnosis	222	76.6%
	Recurrence	49	16.9%
	Distant metastasis	16	5.6%
	Recurrent metastasis	1	0.3%
Prior Resection at an Outside Institution (*n* = 290)	Yes	81	28%
	No	209	72%
R-Status at Outside Resection (*n* = 76)	R0	7	9%
	R1	44	58%
	R2	15	20%
	RX	10	13%
R-Status of Resection at Internal Institution (*n* = 280)	R0	241	86%
	R1	22	7.9%
	R2	13	4.7%
	RX	4	1.4%

**Table 3:** Univariate Cox regression of cumulative survival according to Figure 1. HR = Hazard Ratio, CI = Confidence Interval. * *p* ≤ 0.05.

Characteristics	HR (95% CI)	*p* Value
Metastasis, M1 vs. M0	0.780 (0.636–0.957)	0.017 *
Grading, G1 vs. G2	0.235 (0.068–0.808)	0.022 *
Grading, G1 vs. G3	0.148 (0.045–0.483)	0.002 *
Amputation, no vs. yes	0.242 (0.123–0.478)	0.000 *

**Table 4:** Characteristics of four different groups of closure/reconstruction techniques used. * indicates statistical significance (*p* ≤ 0.05). ^a^ group of split skin graft vs. group of primary closure; ^b^ local/regional flap vs. primary closure; ^c^ free flap vs. primary closure.

Closure/Reconstruction	Primary Closure (*n* = 168)	Split Skin Graft (*n* = 22)	Local/Regional Flap (*n* = 54)	Free Flap with Microvascular Anastomosis (*n* = 40)
Median Age (at Timepoint of Reconstruction/Closure)				
Median (Area)	56.1 (7.4–89.1)	67.5 (28.9–89.1)	57.4 (15.2–86.7)	56.7 (21.25–86.1)
*p*-value (Fisher’s Exact Test)		0.01 *^a^	0.99 ^b^	0.86 ^c^
Sex				
Men	53.6%	59.1%	61.1%	67.5%
Women	46.4%	40.9%	38.9%	32.5%
*p*-value (Fisher´s Exact Test)		0.657 ^a^	0.035 *^b^	0.155 ^c^
Grading				
G1	25%	-	13.5%	6.5%
G2	31.1%	50%	37.8%	48.4%
G3	43.9%	50%	48.6%	45.2%
*p*-value (Fisher´s Exact Test), G1-G3		0.111 ^a^	0.218 ^b^	0.055 ^c^
*p*-value (Fisher´s Exact Test), G1 vs. G2				0.035 *^c^
R-Status				
R0	79%	95.2%	88.9%	97.5%
R1	9.9%	-	9.3%	2.9%
R2	8.0%	4.8%	1.9%	-
RX	3.1%	-	-	-
*p*-value (Fisher´s Exact Test), R0-RX		0.462 ^a^	0.258 ^b^	0.108 ^c^
*p*-value (Fisher´s Exact Test), R0				0.02 *^c^
Size of Excision				
≤ 125 cm³	18.4%	52.4%	32.7%	35.3%
126–1000 cm³	37.5%	38.1%	30.8%	26.5%
1000–10.000 cm³	35.3%	9.5%	36.5%	32.4%
> 10.000 cm³	8.8%	-	-	5.9%
*p*-value (Fisher´s Exact Test)		0.003 *^a^	0.028 *^b^	0.219 ^c^
Recurrence/Metastasis Status at Timepoint of Resection				
First Diagnosis	72%	86.4%	81.5%	80%
Recurrence	16.1%	9.1%	18.5%	20%
Metastasis	8.9%	-	-	-
Suspicion of Recurrence/Metastasis with Neoadjuvant RT/CT	2.4%	4.5%	-	-
Re-Metastasis	0.6%	-	-	-
*p*-value (Fisher´s Exact Test)		0.328 ^a^	0.098 ^b^	0.252 ^c^

**Table 5:** Analysis of the rates of major complications. Major complications after plastic reconstruction are defined as complications that are directly linked to plastic reconstruction; major complications of sarcoma resection are defined as complications that are not linked to plastic reconstruction; and total major complications are defined as the sum of the two. * indicates statistical significance (*p* ≤ 0.05). ^a^ group of split skin graft vs. group of primary closure; ^b^ local/regional flap vs. primary closure; ^c^ free flap vs. primary closure; ^d^ local/regional flap vs. group of split skin graft; ^e^ free flap vs. group of split skin graft.

Number of Complications	Primary Closure (*n* = 168)	Split Skin Graft (*n* = 22)	Local/Regional Flap (*n* = 54)	Free Flap with Microvascular Anastomosis (*n* = 40)
Total Major Complications				
No	90.5%	68.2%	59.3%	70%
1	6.0%	22.7%	29.6%	17.5%
2	1.2%	9.1%	7.4%	7.5%
≥3	2.4%	-	3.7%	5%
*p*-value (Fisher´s Exact Test)		0.007 *^a^	0.001 *^b^	0.004 *^c^
*p*-value (Fisher´s Exact Test)			0.861 ^d^	0.844 ^e^
Major Complications Sarcoma Resection				
No	90.4%	72.7%	61.1%	70%
1	6%	22.7%	29.6%	17.5%
2	1.8%	-	5.6%	10%
≥3	1.8%	4.5%	3.7%	2.5%
*p*-value (Fisher´s Exact Test)		0.592 ^a^	0.544 ^b^	0.639 ^c^
Major Complications Plastic Reconstruction				
No		81.8%	64.8%	70%
1		13.6%	27.8%	20%
2		-	5.6%	7.5%
≥3		4.5%	1.9%	2.5%
*p*-value (Fisher´s Exact Test)			0.290 ^d^	0.599 ^e^

**Table 6:** Analysis of the type of major complication.

Major Complications after Plastic Reconstruction			
Type of Complication	Split Skin Graft (*n* = 22)	Local/Regional Flap (*n* = 54)	Free Flap with Microvascular Anastomosis (*n* = 40)
Hematoma Recipient Region	9.1%	9.3%	2.5%
Hematoma Harvesting Region	-	1.9%	2.5%
Graft/Flap Loss	4.5%	1.9%	10%
Partial Graft/Flap Loss	4.5%	7.4%	17.5%
Arterial Thrombosis	-	-	5%
Venous Thrombosis	-	-	2.5%
Wound-Healing Disorder Recipient	18.2%	22.2%	7.5%
Wound-Healing Disorder Harvesting	-	1.9%	2.5%
Wound Infection	4.5%	-	5.5%
Follow-up Operation (In Progress)			
Additional Local/Regional Flap	-	7.5%	-
Additional Free Flap	22.7%	5.6%	5%

**Table 7:** Major complication rates in relation to the patient’s age.

Age (Years)	0–20 (*n* = 4)	20–40 (*n* = 39)	40–60 (*n* = 92)	60–80 (*n* = 126)	>80 (*n* = 23)
Total Major Complications					
No	100%	87%	80%	78%	74%
1	-	10%	13%	13%	22%
2	-	3%	3%	5%	4%
>3	-	-	3%	4%	-

**Table 8:** Recurrence rate in relation to the different closure/reconstruction methods.

Local Recurrence	Primary Closure (*n* = 168)	Split Skin Graft (*n* = 22)	Local/Regional Flap (*n* = 54)	Free Flap with Microvascular Anastomosis (*n* = 40)
No	81%	77%	93%	95%
Yes	19%	23%	7%	5%

**Table 9:** Results of multivariate analysis on survival using the Cox regression hazard model on recurrence and surgical complications using a Tukey post-hoc test. * indicates statistical significance (*p* ≤ 0.05).

Survival (Cox Regression)			Recurrence (Tukey Post-Hoc Test)			Major Complications (Tukey Post-Hoc Test)		
Charact.	HR (95% CI)	*p* Value	Charact.	F	*p* Value	Charact.	F	*p* Value
Metastasis, M1 vs. M0	2.138 (1.322–3.459)	0.002 *	Grading, G1–3	1; 0.218	0.643	Grading, G1–3	3; 0.162	0.921
Grading, G2 vs. G1	0.587 (0.073–4.387)	0.587	Amputation, yes vs. no	1; 0.218	0.643	Reconstruction Method; Primary Closure, Split Skin Graft, Local/Regional Flap, Free Flap	3; 1.640	0.195
Grading, G3 vs. G1	0.848 (0.112–6.439)	0.874	R-Status, R0-RX	1; 0.132	0.719	R-Status, R0-RX	3; 0.763	0.522
Amputation, yes vs. no	0.798 (0.317–2.007)	0.631	Size of Excision (cm³), ≤125, 126–1000, 1000–10,000, >10,000	1; 0.016	0.901	Size of Excision (cm³), ≤125, 126–1000, 1000–10,000, >10,000	3; 0.200	0.896

## References

[1] Rosenberg S.A., Tepper J., Glatstein E., Costa J., Baker A., Brennan M., DeMoss E.V., Seipp C., Sindelar W.F., Sugarbaker P. (1982). The treatment of soft-tissue sarcomas of the extremities: Prospective randomized evaluations of (1) limb-sparing surgery plus radiation therapy compared with amputation and (2) the role of adjuvant chemotherapy. Ann. Surg..

[2] Harati K., Goertz O., Pieper A., Daigeler A., Joneidi-Jafari H., Niggemann H., Stricker I., Lehnhardt M. (2017). Soft Tissue Sarcomas of the Extremities: Surgical Margins Can Be Close as Long as the Resected Tumor Has No Ink on It. Oncologist.

[3] Rath B., Hardes J., Tingart M., Braunschweig T., Eschweiler J., Migliorini F. (2019). [Resection margins in soft tissue sarcomas]. Orthopade.

[4] Suresh V., Gao J., Jung S.H., Brigman B., Eward W., Erdmann D. (2018). The Role of Reconstructive Surgery After Skeletal and Soft Tissue Sarcoma Resection. Ann. Plast. Surg..

[5] Dadras M., Koepp P., Wallner C., Wagner J.M., Sogorski A., Lehnhardt M., Harati K., Behr B. (2020). Predictors of oncologic outcome in patients with and without flap reconstruction after extremity and truncal soft tissue sarcomas. J. Plast. Reconstr. Aesthet. Surg..

[6] Zeller J., Kiefer J., Braig D., Winninger O., Dovi-Akue D., Herget G.W., Stark G.B., Eisenhardt S.U. (2019). Efficacy and Safety of Microsurgery in Interdisciplinary Treatment of Sarcoma Affecting the Bone. Front. Oncol..

[7] Saebye C., Amidi A., Keller J., Andersen H., Baad-Hansen T. (2020). Changes in Functional Outcome and Quality of Life in Soft Tissue Sarcoma Patients within the First Year after Surgery: A Prospective Observational Study. Cancers.

[8] Elswick S.M., Wu P., Arkhavan A.A., Molinar V.E., Mohan A.T., Sim F.H., Martinez-Jorge J., Saint-Cyr M. (2019). A reconstructive algorithm after thigh soft tissue sarcoma resection including predictors of free flap reconstruction. J. Plast. Reconstr. Aesthet. Surg..

[9] Naghavi A.O., Gonzalez R.J., Scott J.G., Mullinax J.E., Abuodeh Y.A., Kim Y., Binitie O., Ahmed K.A., Bui M.M., Saini A.S. (2016). Implications of staged reconstruction and adjuvant brachytherapy in the treatment of recurrent soft tissue sarcoma. Brachytherapy.

[10] Clavien P.A., Barkun J., de Oliveira M.L., Vauthey J.N., Dindo D., Schulick R.D., de Santibañes E., Pekolj J., Slankamenac K., Bassi C. (2009). The Clavien-Dindo classification of surgical complications: Five-year experience. Ann. Surg..

[11] Restrepo D.J., Huayllani M.T., Boczar D., Sisti A., Spaulding A.C., Moran S.L., Aung T., Bagaria S., Manrique O.J., Forte A.J. (2019). Factors that Influence Chemotherapy Treatment Rate in Patients With Upper Limb Osteosarcoma. Anticancer Res..

[12] Huayllani M.T., Restrepo D.J., Boczar D., Sisti A., Spaulding A.C., Parker A.S., Sarabia-Estrada R., Guerrero-Cazares H., Moran S.L., Forte A.J. (2019). Osteosarcoma of the Upper Extremities: A National Analysis of the US Population. Anticancer Res..

[13] Goertz O., Pieper A., Lohe L.V., Stricker I., Dadras M., Behr B., Lehnhardt M., Harati K. (2020). The Impact of Surgical Margins and Adjuvant Radiotherapy in Patients with Undifferentiated Pleomorphic Sarcomas of the Extremities: A Single-Institutional Analysis of 192 Patients. Cancers.

[14] O’Donnell P.W., Griffin A.M., Eward W.C., Sternheim A., Catton C.N., Chung P.W., O’Sullivan B., Ferguson P.C., Wunder J.S. (2014). The effect of the setting of a positive surgical margin in soft tissue sarcoma. Cancer.

[15] Gundle K.R., Gupta S., Kafchinski L., Griffin A.M., Kandel R.A., Dickson B.C., Chung P.W., Catton C.N., O’Sullivan B., Ferguson P.C. (2017). An Analysis of Tumor- and Surgery-Related Factors that Contribute to Inadvertent Positive Margins Following Soft Tissue Sarcoma Resection. Ann. Surg. Oncol..

[16] Stojadinovic A., Leung D.H., Hoos A., Jaques D.P., Lewis J.J., Brennan M.F. (2002). Analysis of the prognostic significance of microscopic margins in 2,084 localized primary adult soft tissue sarcomas. Ann. Surg..

[17] Novais E.N., Demiralp B., Alderete J., Larson M.C., Rose P.S., Sim F.H. (2010). Do surgical margin and local recurrence influence survival in soft tissue sarcomas?. Clin. Orthop. Relat. Res..

[18] Trovik C.S., Bauer H.C., Alvegård T.A., Anderson H., Blomqvist C., Berlin O., Gustafson P., Saeter G., Wallöe A. (2000). Surgical margins, local recurrence and metastasis in soft tissue sarcomas: 559 surgically-treated patients from the Scandinavian Sarcoma Group Register. Eur. J. Cancer.

[19] Potter B.K., Hwang P.F., Forsberg J.A., Hampton C.B., Graybill J.C., Peoples G.E., Stojadinovic A. (2013). Impact of margin status and local recurrence on soft-tissue sarcoma outcomes. J. Bone Joint Surg. Am..

[20] Zagars G.K., Ballo M.T., Pisters P.W., Pollock R.E., Patel S.R., Benjamin R.S., Evans H.L. (2003). Prognostic factors for patients with localized soft-tissue sarcoma treated with conservation surgery and radiation therapy: An analysis of 1225 patients. Cancer.

[21] Ahmad R., Jacobson A., Hornicek F., Haynes A.B., Choy E., Cote G., Nielsen G.P., Chen Y.L., DeLaney T.F., Mullen J.T. (2016). The Width of the Surgical Margin Does Not Influence Outcomes in Extremity and Truncal Soft Tissue Sarcoma Treated With Radiotherapy. Oncologist.

[22] Traub F., Griffin A.M., Wunder J.S., Ferguson P.C. (2018). Influence of unplanned excisions on the outcomes of patients with stage III extremity soft-tissue sarcoma. Cancer.

[23] Mesko N.W., Wilson R.J., Lawrenz J.M., Mathieu J.L., Ghiam M.K., Mathis S.L., Halpern J.L., Schwartz H.S., Holt G.E. (2018). Pre-operative evaluation prior to soft tissue sarcoma excision—Why can’t we get it right?. Eur. J. Surg. Oncol..

[24] Serletti J.M., Carras A.J., O’Keefe R.J., Rosier R.N. (1998). Functional outcome after soft-tissue reconstruction for limb salvage after sarcoma surgery. Plast. Reconstr. Surg..

[25] Leckenby J.I., Deegan R., Grobbelaar A.O. (2018). Complex Reconstruction After Sarcoma Resection and the Role of the Plastic Surgeon: A Case Series of 298 Patients Treated at a Single Center. Ann. Plast. Surg..

[26] Cordeiro P.G., Neves R.I., Hidalgo D.A. (1994). The role of free tissue transfer following oncologic resection in the lower extremity. Ann. Plast. Surg..

[27] Barner-Rasmussen I., Popov P., Böhling T., Tarkkanen M., Sampo M., Tukiainen E. (2009). Microvascular reconstruction after resection of soft tissue sarcoma of the leg. Br. J. Surg..

[28] Popov P., Tukiainen E., Asko-Seljaavaara S., Huuhtanen R., Virolainen M., Virkkunen P., Blomqvist C. (2000). Soft tissue sarcomas of the lower extremity: Surgical treatment and outcome. Eur. J. Surg. Oncol..

[29] Evans G.R., Black J.J., Robb G.L., Baldwin B.J., Kroll S.S., Miller M.J., Reece G.P., Schusterman M.A. (1997). Adjuvant therapy: The effects on microvascular lower extremity reconstruction. Ann. Plast. Surg..

[30] Reece G.P., Schusterman M.A., Pollock R.E., Kroll S.S., Miller M.J., Baldwin B.J., Romsdahl M.M., Janjan N.A. (1994). Immediate versus delayed free-tissue transfer salvage of the lower extremity in soft tissue sarcoma patients. Ann. Surg. Oncol..

[31] Tejani M.A., Galloway T.J., Lango M., Ridge J.A., von Mehren M. (2013). Head and neck sarcomas: A comprehensive cancer center experience. Cancers.

[32] Khansa I., Janis J.E. (2015). Modern reconstructive techniques for abdominal wall defects after oncologic resection. J. Surg. Oncol..

[33] Cannon C.P., Ballo M.T., Zagars G.K., Mirza A.N., Lin P.P., Lewis V.O., Yasko A.W., Benjamin R.S., Pisters P.W. (2006). Complications of combined modality treatment of primary lower extremity soft-tissue sarcomas. Cancer.

[34] López J.F., Hietanen K.E., Kaartinen I.S., Kääriäinen M.T., Pakarinen T.K., Laitinen M., Kuokkanen H. (2015). Primary flap reconstruction of tissue defects after sarcoma surgery enables curative treatment with acceptable functional results: A 7-year review. BMC Surg..

[35] Kang S., Han I., Kim S., Lee Y.H., Kim M.B., Kim H.S. (2014). Outcomes after flap reconstruction for extremity soft tissue sarcoma: A case-control study using propensity score analysis. Eur. J. Surg. Oncol..

[36] Cassidy R.J., Indelicato D.J., Gibbs C.P., Scarborough M.T., Morris C.G., Zlotecki R.A. (2016). Function Preservation After Conservative Resection and Radiotherapy for Soft-tissue Sarcoma of the Distal Extremity: Utility and Application of the Toronto Extremity Salvage Score (TESS). Am. J. Clin. Oncol..

[37] Harriman S.L., Patel J. (2016). When are clinical trials registered? An analysis of prospective versus retrospective registration. Trials.

[38] Viñals J.M., Rodrigues T.A., Sildenikova D.P., Payro J.M., Porté J.A., Suñe C.H., Ojeda A.L., Vidal J.M., Dewever M., Lopez C.C. (2012). Indications of microsurgery in soft tissue sarcomas. J. Reconstr. Microsurg..

[39] Koulaxouzidis G., Simunovic F., Bannasch H. (2016). Soft Tissue Sarcomas of the Arm—Oncosurgical and Reconstructive Principles within a Multimodal, Interdisciplinary Setting. Front. Surg..

[40] Slump J., Hofer S.O.P., Ferguson P.C., Wunder J.S., Griffin A.M., Hoekstra H.J., Bastiaannet E., O’Neill A.C. (2018). Flap reconstruction does not increase complication rates following surgical resection of extremity soft tissue sarcoma. Eur. J. Surg. Oncol..

[41] Götzl R., Sterzinger S., Semrau S., Vassos N., Hohenberger W., Grützmann R., Agaimy A., Arkudas A., Horch R.E., Beier J.P. (2019). Patient’s quality of life after surgery and radiotherapy for extremity soft tissue sarcoma—A retrospective single-center study over ten years. Health Qual. Life Outcomes.

[42] Slump J., Bastiaannet E., Halka A., Hoekstra H.J., Ferguson P.C., Wunder J.S., Hofer S.O.P., O’Neill A.C. (2019). Risk factors for postoperative wound complications after extremity soft tissue sarcoma resection: A systematic review and meta-analyses. J. Plast. Reconstr. Aesthet. Surg..

[43] Lawrenz J.M., Mesko N.W., Marshall D.C., Featherall J., George J., Gordon J., Vijayasekaran A., Nystrom L.M., Schwarz G.S. (2020). Immediate Versus Staged Soft Tissue Reconstruction After Soft Tissue Sarcoma Resection Has Similar Wound and Oncologic Outcomes. Ann. Plast. Surg..

